# Railway Track Fault Detection Using Selective MFCC Features from Acoustic Data

**DOI:** 10.3390/s23167018

**Published:** 2023-08-08

**Authors:** Furqan Rustam, Abid Ishaq, Muhammad Shadab Alam Hashmi, Hafeez Ur Rehman Siddiqui, Luis Alonso Dzul López, Juan Castanedo Galán, Imran Ashraf

**Affiliations:** 1 School of Computer Science, University College Dublin, D04 V1W8 Dublin, Ireland; furqan.rustam1@gmail.com; 2 Department of Computer Science & Information Technology, The Islamia University of Bahawalpur, Bahawalpur 63100, Pakistan; abid.ishaq@iub.edu.pk; 3 Faculty of Computer Science and Information Technology, Khawaja Fareed University of Engineering and Information Technology, Rahim Yar Khan 64200, Pakistan; shadab.alam@kfueit.edu.pk (M.S.A.H.); hafeez@kfueit.edu.pk (H.U.R.S.); 4 Research Group on Food, Nutritional Biochemistry, and Health, Universidad Europea del Atlántico, Isabel Torres 21, 39011 Santander, Spain; juan.castanedo@uneatlantico.es; 5 Department of Projects, Universidad Internacional Iberoamericana, Campeche 24560, Mexico; 6 Fundación Universitaria Internacional de Colombia, Bogotá 11001, Colombia; 7 Universidad Internacional Iberoamericana, Arecibo, PR 00613, USA; 8 Department of Projects, Universidade Internacional do Cuanza, Cuito EN250, Bié, Angola; 9 Department of Information and Communication Engineering, Yeungnam University, Gyeongsan 38541, Republic of Korea

**Keywords:** vehicle automation, railway track fault detection, mel frequency cepstral coefficient, acoustic data, machine learning

## Abstract

Railway track faults may lead to railway accidents and cause human and financial loss. Spatial, temporal, and weather elements, and wear and tear, lead to ballast, loose nuts, misalignment, and cracks leading to accidents. Manual inspection of such defects is time-consuming and prone to errors. Automatic inspection provides a fast, reliable, and unbiased solution. However, highly accurate fault detection is challenging due to the lack of public datasets, noisy data, inefficient models, etc. To obtain better performance, this study presents a novel approach that relies on mel frequency cepstral coefficient features from acoustic data. The primary objective of this study is to increase fault detection performance. As well as designing an ensemble model, we utilize selective features using chi-square(chi2) that have high importance with respect to the target class. Extensive experiments were carried out to analyze the efficiency of the proposed approach. The experimental results suggest that using 60 features, 40 original features, and 20 chi2 features produces optimal results both regarding accuracy and computational complexity. A mean accuracy score of 0.99 was obtained using the proposed approach with machine learning models using the collected data. Moreover, this performance was significantly better than that of existing approaches; however, the performance of models may vary in real-world settings.

## 1. Introduction

The railway industry has been considered the backbone of a country’s economy, transporting goods and people, and thus offering a potential share in the development of a country. In contrast to road vehicles, trains carry a larger number of people which makes them attractive both to governments and the general population. The public has a low tolerance level for train accidents as they involve a high risk of damage to humans, as well as substantially influencing economic activities. Such accidents put a country’s reputation at risk and political and social risk levels can rise [1]; however, avoiding or reducing the frequency of such accidents to a minimum is very challenging. Derailment, injury, economic burden, death, and loss of public confidence are all undesirable consequences of railway track defects and failure. During railway track maintenance and inspection activities, maintenance staff can also receive injuries or lose their lives [2]. Thus, safe railway operations need proper maintenance, which significantly relies on railway track inspection and fault detection [1]. The safety, reliability, and cost-effectiveness of railway operations are all dependent on railway track condition monitoring. Governments also set regulations for frequent railway track inspections, which generally require a lot of manpower and resources. Therefore, railway track condition monitoring and fault detection are critical due to safety, regulatory, and economic factors [3,4].

Every year a large number of people in Pakistan travel by train. From 2018 to 2019 approximately 70 million people used rail to reach their destinations [5]. Pakistan railway freight also transported 7.4 million tonnes in the year 2020 [6]. However, in the past few years, several serious accidents took place that caused huge human and economic losses [7]. Such accidents can happen due to human error, weather conditions, or faulty railway tracks. According to the Pakistan railway’s annual statistics, train derailments due to railway track faults caused 127 accidents between 2013 to 2020 [8]. The year 2014 was the worst year for the Pakistan railway, as 228 freight trains and 16 passenger trains were derailed, the maximum for any state [9]. In 2019, 23 bogies of freight trains were derailed near Sukkur. Importantly most of the train derailments occurred between the Sukkur–Multan sections [10]. The main reason behind this is the poor condition of railway tracks and the lack of modern resources and techniques to monitor track conditions.

Railway systems around the world operate in a variety of environments where the railway track is threatened by temporal, spatial, and weather factors. The presence of cracks and track conditions are the major factors in rail derailment. Manual inspections consume huge resources and time [11]. They are also prone to human bias and judgement errors [12]. An automated method is required to address the issue of derailment and to ensure the proper investigation of tracks. The objective is to create a system that can assess the given inputs and provide a clear indication of whether the track is faulty or not. This study is concerned with creating a reliable system that can analyze sound signals from tracks and detect whether a track is cracked or not. Railways are one of Pakistan’s most important modes of transportation and have recently experienced a series of rail catastrophes. Keeping this in mind, railway tracks are the most important factor in derailment, and an efficient and effective track fault detection system is needed.

Machine-learning- and deep-learning-based systems have achieved good results in a variety of applications due to recent advancements in these techniques [13]. As railway derailment directly affects human life and the economy, this motivated us to design a system to improve the performance of railway track detection using a machine-learning-based approach [14,15]. Image-processing-based approaches are utilized predominantly, along with other sensors, for railway track detection [16]. However, approaches that require dedicated sensors are expensive and methods involving image processing require higher computational processing capabilities. In contrast, we propose a simple yet efficient method using acoustic signals. A customized railway was used to collect the data from railway tracks; the dataset is described in detail in [17]. Experiments were undertaken using seven well-known machine learning models including logistic regression (LR), support vector machine (SVM), Adaboost classifier (ADA), gradient boosting machine (GBM), extra trees classifier (ETC), and k-nearest neighbor (KNN). Random forest (RF), convolutional neural network (CNN), long short-term memory (LSTM) and CNN-LSTM were used as the deep learning models for the experiments.To assess the performance of the models, chi-square (chi2) was used to enlarge the feature set.

The rest of the study is organized as follows: Section 2 describes the railway track fault-related studies. Section 3 describes the dataset and the different algorithms used in the study. Section 4 discusses the results and findings of the study, while the conclusion is given in Section 5.

## 2. Related Work

Machine learning models can be effective for tackling a variety of problems in such areas as computer vision applications [18,19], text mining [20], image processing [21], and the IoT [17,22], etc. This study also used a machine learning approach for railway track fault detection. In manual railway tracking, fault detection is very difficult, time-consuming, and labor-intensive. AI advancements have led to more precise and accurate railway track fault detection systems while dealing with sensitive data. Railway cracks are the leading cause of derailment all around the world. Machine learning and deep learning models have been proposed to identify these.

Shafique et al. [17] used an acoustic analysis approach to design an automatic railway track fault detection system. They collected data using the traditional railway cart system. Due to their common occurrence, they considered three types of tracks including normal tracks, wheel burnt tracks, and superelevated tracks. They used several machine learning models and showed that RF and DT were able to achieve 97% accuracy. Similarly, by using acoustic analysis, Bhushan et al. [23] proposed a system for the early detection and diagnosis of faults in railway points. An NS-AM-type railway point machine with audio sensors was used for collection of the dataset. This study mainly analyzed faults such as slackened nuts, ballast blast obstruction, and ice obstruction. Two experiments were conducted, one for fault classification on the whole dataset and the other for fault classification. The model evaluation showed an accuracy of 94.1%. Hashmi et al. [24] proposed a conventional acoustic-based system for automatic railway fault detection. They used deep learning models including CONV1D, CONV2D, recurrent neural networks (RNN), and LSTM to address the problem. They considered three types of faults including normal tracks, wheel-burnt tracks, and superelevated tracks. Audio samples of different duration were used to analyze the performance of each model. Each 17 s audio sample was divided into three segments of 1.7 s, 3.4 s, and 8.5 s; the deep learning models were trained and tested against each segment. The performance of the models was investigated using various combinations of audio data augmentation. For the 8.5 s segment, LSTM achieved an accuracy of 99.7%.

Predominantly, image-processing-based methods are utilized for railway track fault detection. For example, Ritika et al. [25] proposed a computer-vision-based system for real-time railway track fault detection. They used a camera mounted on a locomotive to capture images at 30 frames per second. For binary classification, the Inception V3 model was used on the ImageNet dataset. For vegetation overgrowth, the model generalized well on actual vegetation images with a 97.5% precision value. The Sun Kink classifier can professionally classify simulated Sun Kink videos. Similarly, study [26] used different variants of the deep convolutional neural network (DCNN) for railway track fault detection using image data. They used the DCNN-small, DCNN-medium, and DCNN-large networks in their work. The different network architectures were characterized by different sizes and activation functions. The experimental results showed an accuracy of 92% for the large DCNNs.

Manikandan et al. [27] proposed a feed-forward neural network to detect and segment faults from railway track images. They used an adaptive histogram equalization technique to track image enhancement and then features were extracted from the enhanced images. The proposed feed-forward back propagation neural network achieved a 94.9%, 89.99%, and 98.96% accuracy score, sensitivity score, and specificity score, respectively, on the enhanced images. Santur et al. [28] proposed a computer-vision-based system for the inspection of faults in railway tracks. They only inspected faults such as scouring, breaking, and deficient fasteners. The authors extracted the features from video images containing the healthy railway track, while, for the faulty tracks, virtual faults were generated on the original images. Using a modified RF, the highest accuracy of 98% was obtained with HM features.

Tastimur et al. [29] performed fault detection and classification using railway track images with the AdaBoost classifier. Various image processing techniques were also included in their work and they achieved an accuracy of 94.73% for defect detection and 87% for defect classification. Defect detection refers to confirming if there is a defect present while defect classification refers to deciding the type of defect. Chen et al. [30] proposed a deep-learning-based system using B-scan image recognition of rail defects with an improved YOLOV3 algorithm. The proposed system automatically positions a box in B-scan images and recognizes normal bolt holes, EFBWs (electric flash butt wheels), SSCs (shell spallings or corrugation), and BHBs (bolt hold breaks). The experiments involved used 453 B-scan images as a test dataset. The results demonstrated that the improved YOLOV3 achieved a precision of 87.41%. Similarly, Li et al. [31] proposed an ensemble learning model that uses multiple learning algorithms for better predictive performance. They used multiple backbone neural networks individually to obtain the features and mixed them in a binary format to obtain diverse and improved sub-networks. Different image augmentation and feature augmentation techniques were randomly used to achieve diversity. On an 8-defect class dataset, the proposed MBDA (multi backbone double augmentation) system achieved a 2.8% higher mAP.5 compared with faster R-CNN and a 74% higher mAP.5 compared with YOLOV5.

Nandhini et al. [32] used an unsupervised multi-scale CNN for robust automatic railway tracking for detection. They used vibration data for crack detection. They used an open-source dataset in their study. Different machine learning models with different feature extraction techniques were used; the proposed CNN system achieved an accuracy of 89%. A comprehensive overview of the literature shows that current techniques perform well in the detection of faults. Computer-vision-based techniques are extensively used in this regard; acoustic-based techniques still need development for the efficient detection of railway faults. The results obtained indicate that both image-processing- and acoustic-based approaches perform well with respect to railway track fault detection; however, research into the use of acoustic approaches is lacking. Dedicated research efforts are needed in this context. Table 1 provides an analytical overview of the research studies discussed.

**Gaps and Limitations**: In recent years, considerable progress has been made in the railway track fault detection domain. However, there are still several aspects that require further attention. Most of the previous studies have relied on computer vision and image-processing techniques, which can render real-time applications less reliable. Factors such as image quality and weather conditions can significantly impact the accuracy of these approaches. Moreover, the computational cost associated with image-processing techniques is relatively high. Another crucial factor is accuracy. Many of the existing studies exhibit poor accuracy in fault detection, which can be particularly dangerous in applications where accuracy is very important. Therefore, our study aims to address these challenges by focusing on reducing computational costs, employing more reliable fault detection methods using acoustic data methods, and achieving higher accuracy. Through our research, we aim to propose a significant approach that can overcome these limitations and enhance the overall effectiveness of railway track fault detection systems.

## 3. Proposed Methodology

**Figure 1:**
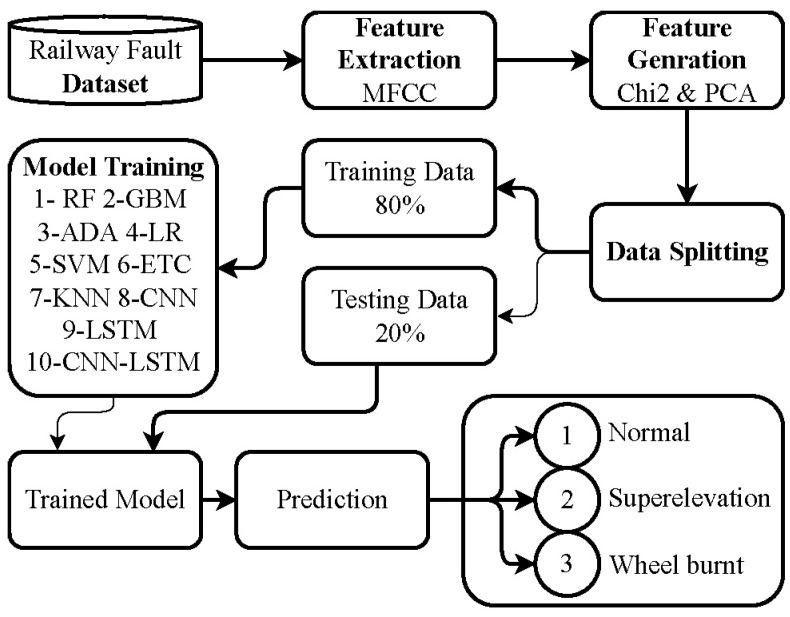
Flow of the proposed methodology.

A supervised machine learning approach to detect faults in railway tracks using aquatic analysis is presented. The methodology for railway fault detection is illustrated in Figure 1. Initially, an acoustic dataset was collected for use in the experiments undertaken. To enable the utilization of audio data in training models, MFCC features were extracted from the audio dataset. These MFCC features capture patterns from the dataset and convert them into a numerical representation, thereby facilitating more effective model training. However, not all of the extracted features in the dataset are equally significant for model training. To address this issue, feature selection techniques were employed. In particular, the chi2 feature selection technique was applied to identify and retain the most significant features. This process involved using the original 40 features and generating 20 new features, which enriched the feature set. Subsequently, the data was divided into training and testing sets with an 80:20 ratio. A total of 80% of the data was used for training the models, while the remaining 20% was used for model testing. The evaluation of the models included metrics such as accuracy, precision, recall, the F1 score and the construction of a confusion matrix to assess their performance.

### 3.1. Dataset

**Figure 2:**
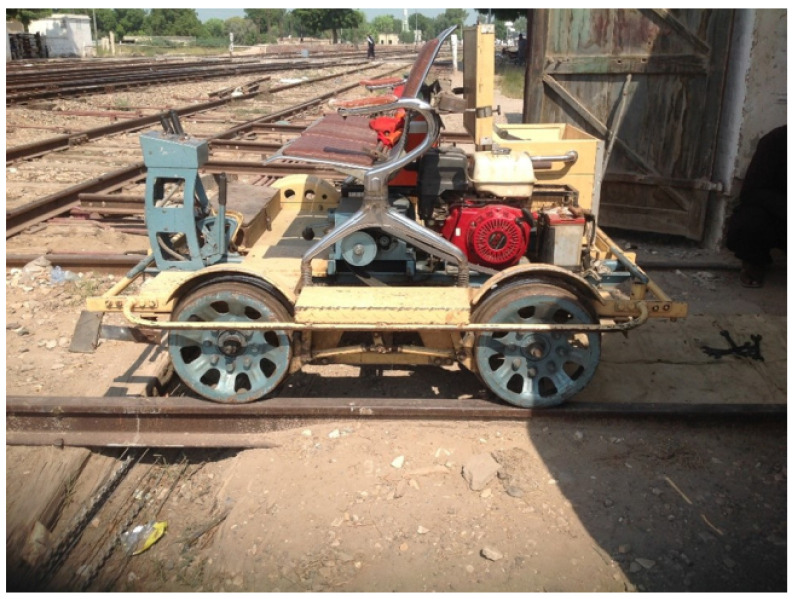
Railway cart used for data collection.

The dataset used in this study was taken from [17], which used a dataset compiled in the Sadiq Abad (Rahim Yar Khan, Punjab) junction area of Pakistan. It contains 720 mono-channel audio ‘wav’ samples. The data collection setup is shown in Figure 2. It comprises two microphones which are installed at a distance of 1.75 inches from the wheel. Data is collected using a mechanical cart that travels at a speed of 35 miles per hour. Two ECM-X7BMP unidirectional electric condensers each with a 3-pole locking mini plug are used, the microphone’s sensitivity is −44.0 ± 3 dB, and the output impedance is 1.2 kw ± 30%. The microphones are unidirectional and connected by a wire. The operating voltage is 5.0 V, the signal-to-noise ratio is 62 dB, and the dynamic range is 88 dB. For further details, the readers are referred to [17].

The dataset consists of three classes, i.e., normal, superelevation, and wheel burnt. In superelevation, the outer rail of a track is elevated above the desired level. Curved tracks usually have higher outer rails than inner rails. In superelevation, an outside rail is raised to a specific level to produce the desired level of positive cant [17]. Wheel burn occurs when a wheel jams or when a locomotive jumps due to imbalanced ballast. Generally, wheel burns occur where gradients are steep or when rain is frequent [17]. Both these faults are associated with a higher probability of railway accidents and need to be periodically corrected.

The dataset is balanced and contains 720 audio recordings (240 for each class), and the length of each sample is 17S. For data collection, a sampling frequency of 22,050 Hz was used. The audio dataset was collected for the experiments; however, it was not used directly. Several types of features can be extracted from this data. This study used the MFCC features from the data to train the machine learning models. A few samples of the MFCC features from the dataset are given in Table 2.

**Figure 3:**
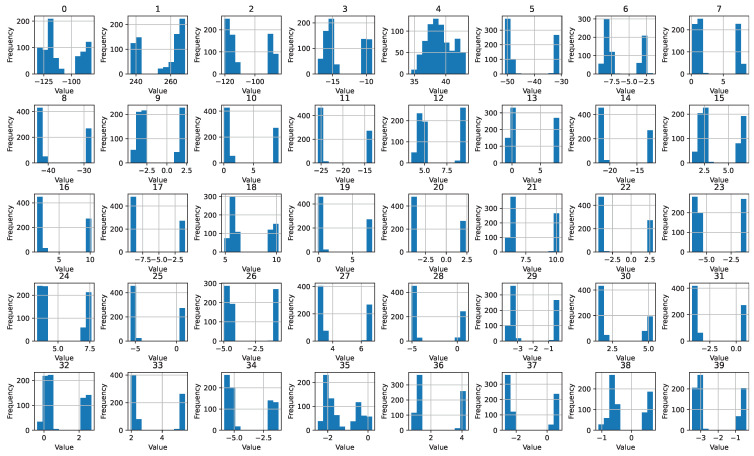
Feature value ranges.

The dataset has 40 features in total with each feature having a different range. Figure 3 shows the feature values and it can be observed that the range of the features, as well as the value of the features, is different, which makes them suitable for classification. However, while the value range may be similar for some features, their threshold is different, which can be used for fault classification.

### 3.2. MFCC (Mel Frequency Cepstral Coefficients)

The proposed system is used for the detection of the three types of railway track faults: normal, wheel burnt, and superelevated. Audio data is used for faulty track detection. Librosa is used for feature extraction (MFCC). This study used 40 MFCC per frame for the audio data. This resulted in a matrix ‘M’ of 758 rows and 40 columns, where the frames are represented by 758 rows and the MFCC values are represented by 40 columns. The following are the steps for implementing the MFCC [33]:

- Shorten the length of the signal by dividing it into short frames.
- For each frame, the estimated power spectrum period gram is calculated.
- For each filter’s total energy, apply the mel-filter bank to the power spectra.
- The filter bank energies are added.
- Take the DCT of the log filter bank energy.
- The first 40 DCT coefficients should be kept, while the rest should be discarded.

**Figure 4:**
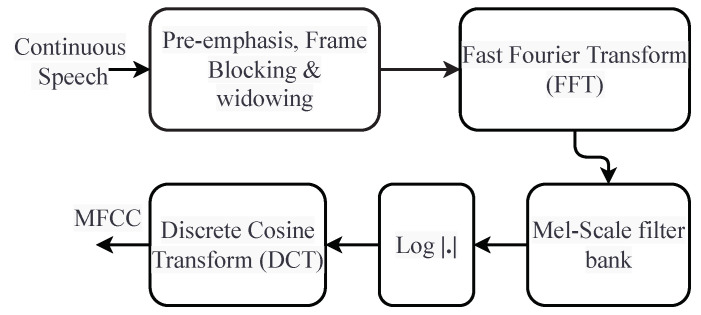
Extraction of MFCC features in five steps [17,34]. The figure explains the methods involved in extracting the MFCC features for use in training and testing the learning models.

MFCC is based on signal disintegration using a filter bank. MFCC produces a discrete cosine transform (DCT) of a real logarithm of short-term energy on the mel frequency scale. The process of extracting the MFCC features is shown in Figure 4.

Equation (1) can be used to express the mel approximation from the physical frequency [35]. The mel for a frequency is calculated as follows:

(1) $$mel\left(f\right)=2592\times \log_{10}\left(1+\frac{1}{700}\right)$$

where the frequency *f* is in Hz and the frequency mel(f) is in mels. The resultant feature vector space *F* of size 40 is obtained as follows:

(2) $$F=[\frac{1}{N}\sum_{i=1}^{758}a_{i1},\frac{1}{N}\sum_{i=1}^{758}a_{i2},\frac{1}{N}\sum_{i=1}^{758}a_{i3},...,\frac{1}{N}\sum_{i=1}^{758}a_{i40}]$$

where *N* is the total number of frames, which is 758 in this study, and *i* is the *i*th frame. The *F* value for all the audio recordings (wheel burnt, superelevated, and normal track) was computed and after that was manually labeled in the dataset, and *F* was then used in the experimentation setup.

(3) $$M={\left(\begin{array}{ccccc} a_{11} & a_{12} & a_{13} & \cdots & a_{1C}\\ a_{21} & a_{22} & a_{23} & \cdots & a_{2C}\\ \vdots & \vdots & \vdots & \vdots & \vdots \\ a_{(R-1),1} & a_{(R-1),2} & a_{(R-1),3} & \cdots & a_{(R-1),C}\\ a_{R1} & a_{R2} & a_{R3} & \cdots & a_{RC} \end{array}\right)}_{RXC}$$

where *C* represents the number of columns and *R* is the number of rows. The rows represent different frames and the columns represent the individual MFCC coefficients, while $a_{ij}$ is the MFCC coefficient value of the *i*th frame and the *j*th MFCC coefficient value.

MFCC uses the quasi-logarithmic spaced frequency scale that closely resembles the human auditory system. The matrix *M* represents the features after performing all the steps shown in Figure 4, and the matrix *M* is used to classify the sample into one of the categories addressed in this study. The matrix *M* contains the extracted MFCC features for a single sample, which means that every sample of railway track crack (normal, wheel burnt, etc.) has its own matrix *M*. Every element of the matrix *M* shows an MFCC coefficient value for a certain frame from a specific crack class. The learning models are both trained and tested using these features.

### 3.3. Chi Square

Chi2 is a well-known and commonly used feature selection technique. It is specially designed for testing the relationship between categorical variables. Chi2 is used to estimate the lack of independence between variables/features in a dataset as well as to compare the chi2 distribution with one degree of freedom to judge extremeness [36]. Chi2 is used for two types of tests: a test for the goodness of fit and a test for independence. The test for independence was used for the feature selection and the dependency of the target label was examined for the features. The correlation of the features can be efficiently investigated through chi2. The features that correlate are kept and the remaining features are discarded. For every feature, chi2 is computed independently towards the target class, and, based on a predefined threshold, its significance is decided. The greater the value of chi2 the less the significance of the features and vice versa. The formula for feature selection in chi2 is represented as

(4) $$X_{c}^{2}=\sum \frac{{\left(O_{i}-E_{i}\right)}^{2}}{E_{i}}$$

where *c* is the degree of freedom (threshold value), *O* shows the observed value, *E* is the expected value, and $X^{2}$ is the chi2 computed value for the features.

**Figure 5:**
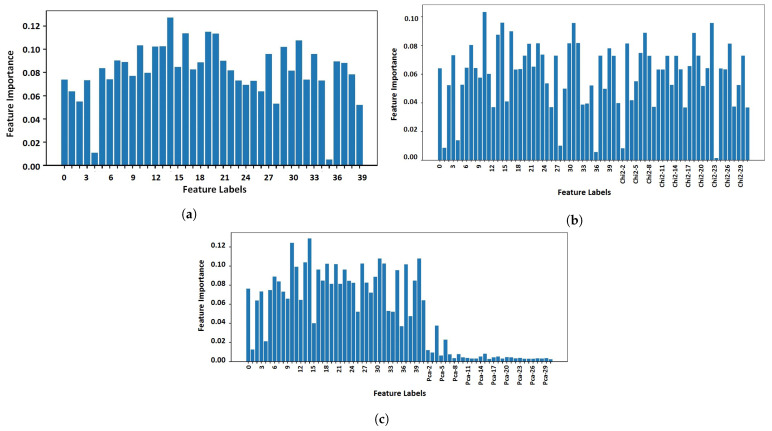
Feature importance comparison generated by PCA and chi2. (**a**) Original features. (**b**) Chi2-generated features. (**c**) PCA-generated features.

In this study, we used chi2 features because it is more efficient compared to other techniques for our dataset. For corroboration, we also utilized the features from principal component analysis (PCA). Figure 5 shows a comparison between the PCA- and chi2-generated features’ importance. We used the extra trees classifier to find the features’ importance. We fitted the model on the dataset by feeding the features and the target. In response, the model found the importance of each feature to accurately predict the target class [37].

### 3.4. Machine Learning Models

This study used several models including LR, RF, SVM, etc. Several important hyperparameters were fine-tuned to improve the performance of the models. The Sci-kit Learn library was used for the implementation of these algorithms. A list of all the hyperparameters used for the experiments is provided in Table 3.

#### 3.4.1. Logistic Regression

LR is a predictive analysis algorithm and statistical method that works on the concept of probability. LR is a supervised learning model and is extensively used to analyze binary data in which one or more variables work together to obtain the final result [38]. LR works well on linearly separable data. LR creates a connection among categorical dependent variables and one or more independent variables by approximation probability using a logistic regression sigmoid function [39]. For the probability, a sigmoid function is used.

#### 3.4.2. Random Forest

RF is a tree-based ensemble model that provides accurate predictions by combining many weak learners. Initially, RF creates multiple decision trees using random features to create a forest. After that, the final prediction is made by combining all the decision trees [40]. Decision tree votes with low error rates are given higher weights and vice versa [41]. The likelihood of a wrong prediction is reduced by using a decision tree with low error rates. RF is usually used to analyze binary data.

#### 3.4.3. K-Nearest Neighbor

KNN is used for both classification and regression problems. KNN is a simple and widely used machine learning algorithm. KNN assumes that similar data can be found nearby so it employs the idea of neighbors. KNN uses distance calculation metrics, such as the Minkowski distance, the Manhattan distance, and the Euclidean distance, to estimate the distance between the new data points to their neighbors. The number of neighbors to consider for the prediction in KNN is determined by the value of *K* [42].

#### 3.4.4. Support Vector Machine

SVM is a well-known machine learning algorithm that is widely used for both linear and nonlinear data classification. Many researchers use SVM for binary classification problems due to the availability of various kernel functions. The primary purpose of SVM is to classify data points by estimating the hyperplane using a feature set [43]. Hyperplane dimensions vary with the number of features. A hyperplane in n-dimensional space has multiple possibilities. The goal is to find the hyperplanes that maximize the margins between the class samples. The cost function is used for the determination of hyperplanes.

#### 3.4.5. Adaboost Classifier

ADA is another ensemble learning classifier that employs a boosting method for weak-learner training (decision trees). Adaptable boosting is the basis of Ada-boost. ADA is the most well-known and widely used algorithm since it was the first to adjust weak learners. Many weak learners are combined by ADA and trained repeatedly on copies of the original dataset, while all weak learners focus on the difficult data facts or outliers. It is a meta-model that takes numerous weak-learner copies and trains them with the same feature set but with various values assigned to them. It is an ensemble model like RF, but it employs a boosting method to ensemble learning models together.

#### 3.4.6. Extra Tree Classifier

ETC works in a similar way to the RF classifier but instead of a top-down approach for splitting, ETC uses a randomized technique, which helps to reduce variance by increasing the tree bias. This is due to the optimal cut-point choice, which is responsible for a large amount of the induced tree’s variance. Unlike the RF, ETC does not use bootstrap copies. Instead, ETC uses the entire learning sample. From a statistical perspective, this concept provides a benefit in terms of split, increasing the bias, although split-point randomization frequently results in great variance reduction [44]. The probabilities of all classes are averaged for prediction, and the class with the highest probability is selected. This complexity reduction helps ETC to produce an improved result in a variety of high-dimensional complex problems and also reduces the computational burden.

#### 3.4.7. Gradient Boosting Classifier

To perform classification problems, GBM employs a boosting technique. GBM is fitted with a large number of weak learners (decision trees) that are trained sequentially on the first classifier errors. The initial decision tree classifier fits the dataset, while subsequent decision trees train on the first classifier’s errors and add to the first, and so on [45]. This method of sequential coupling of classifiers reduces error and improves accuracy. A mean square error (MSE) is defined by GBM.

### 3.5. Deep Learning Models

In addition to machine learning classifiers, deep learning models, such as LSTM and CNN, are also used to detect railway track faults.

#### 3.5.1. Convolutional Neural Network

CNN is a deep neural network that manages the computational complexity of large-size datasets. CNN is a powerful neural network model that uses convolution, dropout, pooling, activation, and non-linear layers to learn complex features. CNN uses an end-to-end approach for the training that makes CNN more efficient [46]. The convolutional layer in CNN is used to extract features. The convolutional operation is shown below

(5) $$x_{j}^{n}=f\left(\sum_{i\epsilon M}w_{ij}^{n}x^{n-1}+b_{j}^{l}\right)$$

where *x* is the *j*th feature of the *n*th layer, f(.) is the activation function, *b* is the offset value that shows the convolution kernel, and *M* is the set of the input feature maps.

The pooling layer is also known as the down-sampling layer and is widely used to reduce the amount of processing by compressing the amount of data and parameters. The activation function in CNN is the rectified linear unit and is calculated as

(6) f(a)=max(x,0)

The softmax function of CNN can be computed using the following

(7) $$fy_{i}=\frac{e^{y_{i}}}{\sum_{j=1}^{n}e^{y_{i}}}$$

#### 3.5.2. Long Short-Term Memory

LSTM is specifically used to address the problem of learning long-term dependencies [47]. The internal architecture of LSTM contains a separate memory cell that can update and expose its content when required. LSTM consists of four gates [48] including the input gate, the forget gate $f_{t}$, the memory cell $c_{t}$, and the output gate $o_{t}$, and a hidden state $h_{t}$. The forget gate determines how much each memory cell unit is erased, the input gate determines how much each unit is updated, and the output gate determines how much internal memory state is exposed. The transition equations for LSTM are the following

(8) $$\begin{array}{cc} \; & i_{t}=\alpha \left(W_{i}X_{t}+U_{i}h_{t}-1+V_{i}C_{t-1}\right)\\ \; & f_{t}=\alpha \left(W_{f}X_{t}+U_{f}h_{t}-1+V_{f}C_{t-1}\right)\\ \; & i_{o}=\alpha \left(W_{o}X_{t}+U_{o}h_{t}-1+V_{o}C_{t-1}\right)\\ \; & {\hat{c}}_{t}=tanh\left(W_{c}X_{t}+U_{c}h_{t}-1\right)\\ \; & c_{t}=f_{t}^{i}Oc_{t-1}+i_{t}O{\hat{c}}_{t}\\ \; & h_{t}=o_{t}Otanh\left(c_{t}\right) \end{array}$$

where α is the logistic sigmoid function and *O* is element-wise multiplication and $x_{t}$ is the input at the current state.

#### 3.5.3. CNN-LSTM Ensemble

Both the LSTM and CNN models are combined sequentially to make the CNN-LSTM model. The architecture of all three used deep learning models is shown in Table 4. All models receive input through the embedding layer, which consists of a 1000 vocabulary size because the value range of the feature set and the output dimension is 100. The 1D Conv layer is used with 64 features and kernel size 3 × 3 in both the CNN and the CNN-LSTM models. The LSTM model is used with 64 recurrent units. All the models are compiled using the categorical_crossentropy loss function and the Adam optimizer. The models are fitted with 100 epochs and 16 batch sizes.

## 4. Results and Discussion

This section contains the results of the machine learning and deep learning models for railway track fault detection. The results reported in previous papers [17,24] are improved in terms of high accuracy and efficiency.

### 4.1. Experiments Using Original Features

Table 5 shows the results of the models using the original features. The evaluation shows that RF, ADA, ETC, and KNN exhibit strong performance, achieving an accuracy score of 0.99. On the other hand, LSTM performs poorly, with an accuracy score of 0.88, as well as underperforming in terms of the other evaluation parameters.

The original feature set comprises only 40 features, making it relatively small. Consequently, the tree-based models, such as RF, ADA, and ETC, show their efficacy for this limited feature set. However, the linear models, such as LR and SVM, and the deep learning models, such as LSTM, CNN, and CNN-LSTM, struggle to achieve significant results on this small feature set. The deep learning models typically require both a larger feature set and a larger number of samples to demonstrate significant performance.

Table 6 shows the results of the models using a 10-fold cross-validation approach, with 10-fold cross-validation. The tree-based models outperform the others as RF and ADA are significant with a mean accuracy score of 0.99 and ±0.01 standard deviation (SD). The deep learning models and the linear models, LR and SVM, are also low in accuracy with 10-fold cross-validation.

**Figure 6:**
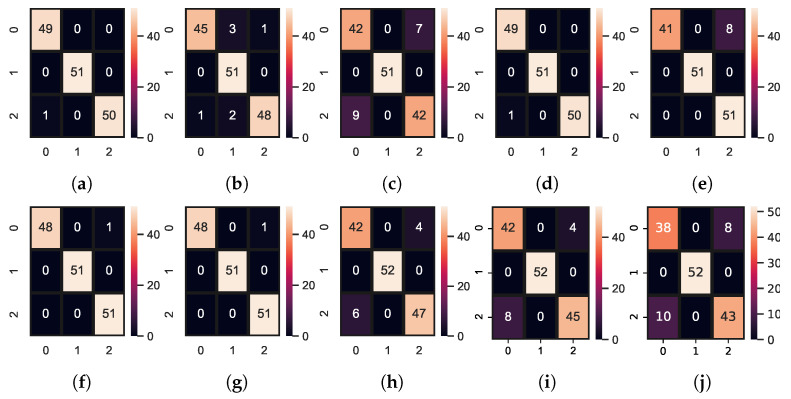
Confusion matrices for machine learning and deep learning models. (**a**) ADA; (**b**) GBM; (**c**) LR; (**d**) RF; (**e**) SVM; (**f**) ETC; (**g**) KNN; (**h**) CNN-LSTM; (**i**) CNN; (**j**) LSTM.

**Figure 7:**
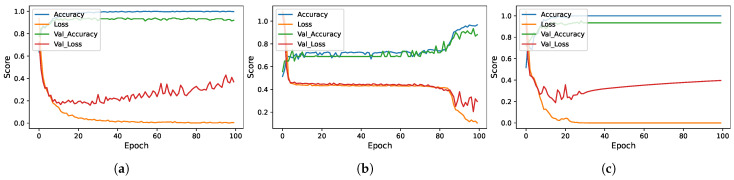
Deep learning models per epoch scores. (**a**) CNN; (**b**) LSTM; (**c**) CNN-LSTM.

We also compared the performance of the models in terms of the number of correct predictions and the number of wrong predictions. Figure 6 shows the results of the learning models in terms of the confusion matrix. RF, KNN ADA, and ETC achieve the best results with the highest number of correct predictions. These models provide 150 correct predictions out of 151 predictions and give only one wrong prediction. Moreover, Figure 7 shows the evaluation parameters score of the models per epoch of the deep learning models.

### 4.2. Impact of Number of Features

Table 7 shows the results of the learning models using 50 features where 10 additional features are generated using the chi2 approach and joined with the original 40 features. With this approach, we increase the number of features to further improve the performance of the learning models. This approach significantly improved the performance of the learning models as RF, ADA, ETC, KNN, and GBM improved accuracy to 1.00. The accuracy of the linear models LR and LSTM was also improved from 0.95 to 0.97 and 0.72 to 0.88, respectively. An increase in the number of features enlarges the feature set which helps the model achieve a better fit and show better performance.

Table 8 shows the performance of models using 10-fold cross-validation with the 50 features dataset. The performance of the models is also improved for the 10-fold case, as LSTM improved its accuracy from 0.74 to 0.87 with ±0.02 SD. CNN and CNN-LSTM also show better performance due to an increase in the feature set size. A higher number of features for training can lead to a better fit for the deep learning models. There was still a gap in terms of accuracy, so we sought to improve this by further increasing the number of features. We generated 10 more features using chi2 and added them to the feature set to make 60 features in total.

Table 9 shows the results of the models using 60 features. Out of 60, 40 features are original and 20 are chi2 generated. The performance of all the models is significantly improved for 60 features in comparison to using 50 features. With the increase in the feature sets, the performance of the deep learning models gradually increases. The deep learning models, especially CNN-LSTM, achieved a significant 0.96 accuracy. The machine learning models also showed significant improvement in terms of all the evaluation parameters.

The 10-fold cross-validation results are shown in Table 10. The models show significant results for the k-fold cross-validation case. When the feature set increases to 60, the performance of all the models is improved. A 1.00 accuracy score with the 60 features dataset was even achieved using the ETC classifier. These results show that chi2 generates the best 20 features from the original 40 features, which helps to achieve a 100% accuracy score. According to the results, as we increase the number of features, the performance also increases. To further ensure the significance of 20 new optimal number of features, 30 features were also generated and experiments were performed.

Table 11 shows the results of the machine learning and deep learning models using the total 70 features dataset. The performance of the models remains the same as with 60 features. There is no significant change in accuracy; instead, a small drop in the performance of the SVM and the deep learning models is observed. As the accuracy did not improve, we considered 60 features for our proposed approach. The results with 70 features are also good, yet the computational cost is higher when using 70 features.

Table 12 shows the results of the machine learning and deep learning models using 10-fold cross-validation. All models show good performance for k-fold cross-validation using 70 features. A larger feature set helps the models to achieve a food fit, which improves their performance. However, no significant improvement is observed when moving from 60 features to 70 features.

### 4.3. Analysis of Feature Space

**Figure 8:**
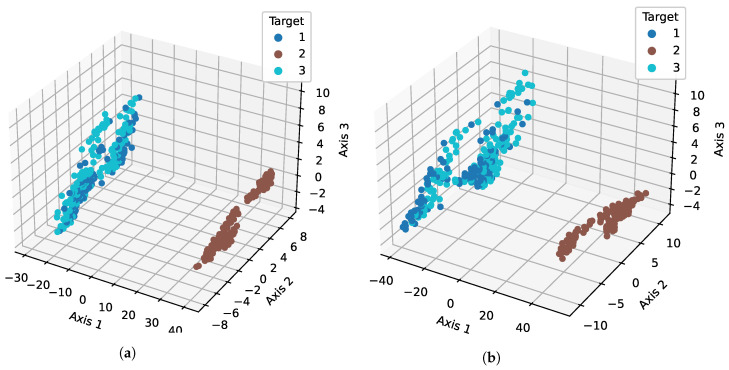
Feature space for original features and chi2-generated 60 feature dataset. We illustrate samples with respect to the target classes. We use the chi2 technique to compress all 60 new features into three dimensions (Axis 1, Axis 2, and Axis 3) and then illustrate it using a 3D scatterplot. We can see that, with the 60 new features, the samples are less overlapped compared to the original set, which helps to improve the accuracy. (**a**) Original features; (**b**) 60 features.

Figure 8 shows the feature space for both the original features and the chi2-generated 60 features. Figure 8a shows that there is overlapping in the target 1 and 3 samples with the original features. However, when additional features from chi2 are added, the overlapping is reduced. The same can be seen in Figure 8b, indicating that the distribution of the class samples becomes more separable.

**Figure 9:**
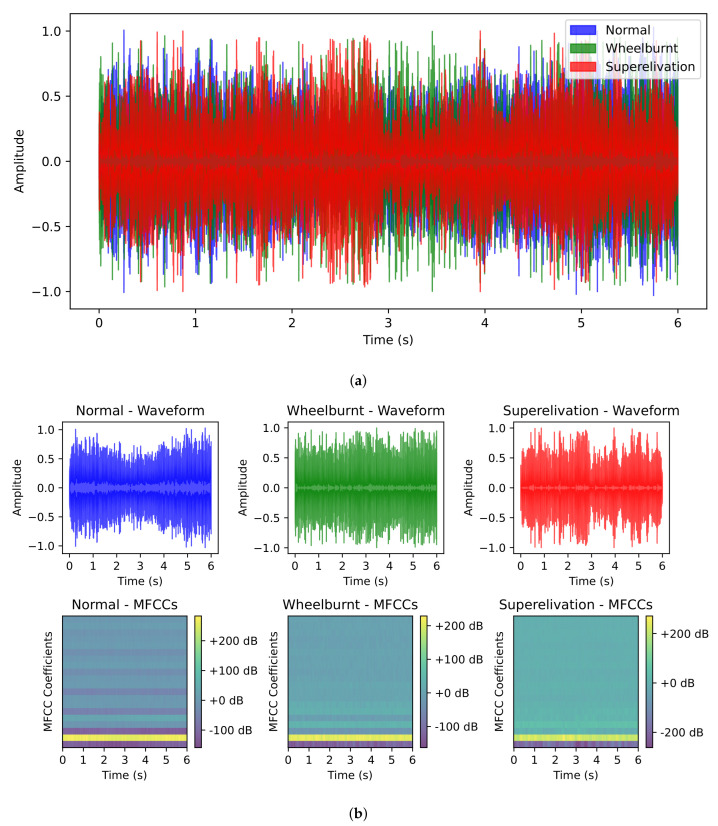
Waveform comparison. (**a**) Overlap of features; (**b**) Distinguishable features.

Additionally, we sought to determine the significance of employing chi2 for feature selection. To achieve this, we visualized the waveforms of the audio samples from each category for comparison, as shown in Figure 9. Through analysis, it became evident that distinct differences exist between the audio features of each category. However, these features exhibit significant overlap with one another, especially in the central region. Figure 9a shows this overlapping, where each category demonstrates different edge points while sharing a common and overlapping center area. By leveraging chi2, we can effectively extract the crucial features from the dataset concerning the target classes and generate a meaningful feature set that enhances the performance of our learning models. All the features are not important with regard to the target class and may share similar values for more than one target class, which causes overlap. Chi2 tests the independence between the features and the target classes and selects those features for which higher dependence is found. So, chi2 eliminates unnecessary features, which also helps to reduce feature overlap, as is the case in this study.

### 4.4. Computational Complexity of Models

As well as the accuracy, the computational time is equally important for the models. The processing time of the models is measured for railway track fault detection. Table 13 shows the computational time of the machine learning and deep learning models for the 40, 50, 60, and 70 feature datasets. It can be observed that as we increase the number of features the computational cost of the models also increases. So, we consider 60 features in the proposed approach to optimize both the accuracy and computational complexity.

We observed that the computational cost of the single LSTM model remained higher than that of the CNN and CNN-LSTM models. Upon further investigation, we found that the difference in the computational cost could be attributed to variations in the number of parameters used in each model. In the LSTM model, we utilized 64 units, whereas in the CNN-LSTM model, we used 32 units with LSTM. This variation in the number of units impacts the computational time for each model. Furthermore, the computational time was found to be system-dependent, with different execution times observed when running the application on different machines. However, the execution time of LSTM remained higher than that for the CNN-LSTM model. The values given in Table 13 indicate averaged values from several runs.

### 4.5. Comparison with Previous Studies

In this section, we compare the proposed approach with previous studies that used the same dataset. The study [17] proposed an approach for fault detection. The authors deployed the RF model on the original feature set extracted using the MFFC technique from the railways’ fault dataset. Similarly, the study [24] proposed an approach for fault detection using the on-the-fly technique. The authors deployed the LSTM model to achieve significant accuracy. In comparison with these studies, we contribute to feature engineering and increase the number of features to improve the accuracy of the state-of-the-art models. Table 14 shows the comparison results for the machine learning and the deep learning models.

### 4.6. Statistical t-Test Analysis

In this section, we present the results of a statistical *t*-test which was performed on the machine learning model results with all the used features. The *t*-test compares two results and shows whether the compared approach is statically significant or not. The *t*-test constructs two hypotheses, which are the null hypothesis and the alternative hypothesis. The null hypothesis is that the compared approach is not statistically significant compared to the others. If the *t*-test rejects the null hypothesis then the alternative hypothesis is accepted, which indicates that the proposed approach is statically significant.

The *t*-test returns output in terms of a T-score and a critical value (CV). If the t-score is greater than the CV then the null hypothesis is rejected. Table 15 shows the results for several scenarios. We compared the machine learning model results using the proposed approach using 60 features with the other features. In all the compared cases, the *t*-test rejects the null hypothesis, indicating that the proposed approach is statistically significant.

## 5. Conclusions

Track status monitoring and fault detection are very important to minimize the risks of railway accidents. The use of acoustic data represents a more efficient and low-resource-requiring solution in this regard. This study employs feature engineering to improve the performance of railway track fault detection. MFCC features are used from acoustic data and the impact of 10, 20, and 30 additional features from chi2 is analyzed with several machine learning and deep learning models. The results suggest that using 60 features, 40 original features, and 20 chi2 features, produces optimal results with respect to both accuracy and computational complexity. A 100% accuracy can be obtained using the proposed approach with machine learning models. The cross-validation results obtained validate this performance. Moreover, this performance is significantly better than that of state-of-the-art approaches. As a result of experimentation, we concluded that the machine learning model’s performance depends on the feature set quality. The optimal number of features helps to improve performance. This study performed experiments on a small dataset, which is not enough for validation purposes, especially for deep learning models, representing a limitation of this study. In the future, we intend to collect more data for railway track faults. We also plan to incorporate the global positioning system for tracking the location of faults.

## Figures and Tables

**Table 1:** Summary of the discussed research works.

Ref.	Results	Models	Dataset	Limitation
[17]	97% accuracy RF and DT	SVM, LR, RF, DT, MLP, CNN	Self-made	Simple state-of-the-art approach
[25]	97.5% precision SunKink	SunKink, inception3	Self-made	High computational cost
[23]	94.1% accuracy	Sensors and GSM module	Self-made	High computational cost and poor performance in terms of accuracy.
[24]	99.7% accuracy LSTM	CONV1D, CONV2D, RNN and LSTM	Shafique et al. [17]	High computational cost because of deep learning approach and spectral features
[26]	92% accuracy DCNN-Large	DCNN-small, DCNN-medium, DCNN-large	Self-made	High computational cost and poor performance
[27]	94.9% Accuracy FFBP	SVM with PCA, Radial NN, FFBP	Self-made	Poor performance in terms of accuracy
[28]	98% accuracy RF with HM features	RF with PCA, RF with KPCA, RF with SVD, RF with HM	Self-made	High computational cost because of vision-based approach
[32]	89% accuracy Multi-scale CNN	Bayes weighted vector, SVM (LDA, PCA), CNN	Kaggle	High computational cost because of deep learning approach
[29]	94.73% accuracy for defect detection, 87% for defect classification	AdaBoost	Self-made	High computational cost because of image processing approach
[30]	87.41% precision	YOLOV3, improved YOLOV3	Self-made	Poor performance whether they used a complex model
[31]	0.75 mAP, WBDA	MBDA, YOLOV5S, YOLOV5S6, YOLOV5m, Faster RCNN R50, Faster RCNN R101	National academy of railway sciences test centre dataset	Poorperformance as model achieved approximately 0.75 accuracy.

**Table 2:** Samples from the original dataset.

1	2	3	…	40	Label
−1.4621756	1.3114967	−2.4462814	…	−3.2169747	1
−0.51381445	4.131112	0.76316893	…	−0.70693094	2
−2.1898634	1.3600227	−2.3395789	…	−3.2751813	3

**Table 3:** Hyperparameters used for machine learning models.

Model	Hyperparameters	Hyperparameters Values Range
LR	solver = ‘saga’, multi_class = ‘multinomial’ C = 3.0	solver = {liblinear, sag, saga}, multi_class = ‘multinomial’ C = {1.0 to 10.0}
SVM	kernel = ‘linear’, C = 3.0	kernel = {linear, sigmoid, poly} C = {1.0 to 10.0}
RF	n_estimators = 200, max_depth = 200, random_state = 2	n_estimators = {10 to 500}, max_depth = {10 to 500}, random_state = {0 to 100}
GBM	n_estimators = 200, max_depth = 200, learning_rat = 0.2	n_estimators = {10 to 500}, max_depth = {10 to 500}, learning_rat = {0.1 to 0.9}
ADA	n_estimators = 200, max_depth = 200, learning_rat = 0.2	n_estimators = {10 to 500}, max_depth = {10 to 500}, learning_rat = {0.1 to 0.9}
ETC	n_estimators = 200, max_depth = 200, random_state = 2	n_estimators = {10 to 500}, max_depth = {10 to 500}, random_state = {0 to 100}
KNN	n_neighbour = 3	n_neighbour = {1 to 5}

**Table 4:** The architecture of the used deep learning models.

CNN	LSTM	CNN-LSTM
Embedding (1000, 100,) Dropout (0.5) Conv1D (64, 3, activation = ‘relu’) MaxPooling1D (pool_size = 3) Flatten () Dense (16) Dense (3, activation = ‘softmax’)	Embedding (1000, 100,) Dropout (0.5) LSTM (64) Dense (32) Dense (3, activation = ‘softmax’)	Embedding (1000, 100,) Dropout (0.5) Conv1D (64, 3, activation = ‘relu’) MaxPooling1D (pool_size = 3) LSTM (32) Dense (16) Dense (3, activation = ‘softmax’)
loss = ‘categorical_crossentropy’, optimizer = ‘adam’, epochs100		

**Table 5:** Results using original features.

Model	Accuracy	Precision	Recall	F1 Score
RF	0.99	0.99	0.99	0.99
GBM	0.95	0.95	0.95	0.95
ADA	0.99	0.99	0.99	0.99
LR	0.89	0.89	0.89	0.89
SVM	0.95	0.95	0.95	0.95
ETC	0.99	0.99	0.99	0.99
KNN	0.99	0.99	0.99	0.99
LSTM	0.88	0.88	0.88	0.88
CNN	0.92	0.92	0.92	0.92
CNN-LSTM	0.93	0.93	0.93	0.93

**Table 6:** 10-fold cross-validation results using original features.

Model	Accuracy	SD
RF	0.99	±0.01
GBM	0.98	±0.02
ADA	0.99	±0.01
LR	0.87	±0.07
SVM	0.94	±0.04
ETC	0.99	±0.01
KNN	0.96	±0.04
LSTM	0.74	±0.02
CNN	0.92	±0.01
CNN-LSTM	0.91	±0.01

**Table 7:** Results using 40 original and 10 chi2 features (50 features).

Model	Accuracy	Precision	Recall	F1 Score
RF	1.00	1.00	1.00	1.00
GBM	1.00	1.00	1.00	1.00
ADA	0.99	0.99	0.99	0.99
LR	0.85	0.85	0.85	0.85
SVM	0.97	0.96	0.96	0.96
ETC	1.00	1.00	1.00	1.00
KNN	1.00	1.00	1.00	1.00
LSTM	0.88	0.87	0.87	0.87
CNN	0.94	0.94	0.94	0.94
CNN-LSTM	0.94	0.94	0.94	0.94

**Table 8:** 10-fold cross-validation results using 40 original and 10 chi2 features (50 features).

Model	Accuracy	SD
RF	0.99	±0.01
GBM	0.98	±0.02
ADA	0.99	±0.01
LR	0.86	±0.07
SVM	0.95	±0.03
ETC	0.99	±0.01
KNN	0.95	±0.04
LSTM	0.87	±0.02
CNN	0.94	±0.01
CNN-LSTM	0.93	±0.01

**Table 9:** Results using 40 original and 20 chi2 features. (60 features).

Model	Accuracy	Precision	Recall	F1 Score
RF	1.00	1.00	1.00	1.00
GBM	0.99	0.99	0.99	0.99
ADA	1.00	1.00	1.00	1.00
LR	0.89	0.89	0.89	0.89
SVM	0.97	0.97	0.97	0.97
ETC	1.00	1.00	1.00	1.00
KNN	1.00	1.00	1.00	1.00
LSTM	0.88	0.87	0.87	0.87
CNN	0.92	0.92	0.92	0.92
CNN-LSTM	0.96	0.95	0.95	0.95

**Table 10:** 10-fold cross-validation results using 40 original and 20 chi2 features (60 features).

Model	Accuracy	SD
RF	0.99	±0.01
GBM	0.98	±0.02
ADA	0.99	±0.01
LR	0.86	±0.06
SVM	0.91	±0.03
ETC	1.00	±0.01
KNN	0.95	±0.04
LSTM	0.87	±0.02
CNN	0.93	±0.01
CNN-LSTM	0.95	±0.01

**Table 11:** Results using 40 original and 20 chi2 features. (70 features).

Model	Accuracy	Precision	Recall	F1 Score
RF	0.99	0.99	0.99	0.99
GBM	0.97	0.97	0.97	0.97
ADA	1.00	1.00	1.00	1.00
LR	0.88	0.88	0.88	0.88
SVM	0.92	0.92	0.92	0.92
ETC	1.00	1.00	1.00	1.00
KNN	1.00	1.00	1.00	1.00
LSTM	0.87	0.87	0.87	0.87
CNN	0.91	0.91	0.91	0.91
CNN-LSTM	0.93	0.93	0.93	0.93

**Table 12:** 10-fold cross-validation results using 40 original and 30 chi2 features (70 features).

Model	Accuracy	SD
RF	0.99	±0.01
GBM	0.97	±0.01
ADA	0.99	±0.01
LR	0.87	±0.03
SVM	0.90	±0.02
ETC	1.00	±0.01
KNN	0.99	±0.01
LSTM	0.87	±0.02
CNN	0.91	±0.01
CNN-LSTM	0.92	±0.01

**Table 13:** Processing time in seconds for each feature set.

Model	Number of Features			
	40	50	60	70
RF	1.04	1.47	2.01	2.11
GBM	2.82	3.47	3.59	4.01
ADA	2.03	2.39	2.51	2.48
LR	0.18	0.22	0.49	0.48
SVM	0.31	0.34	1.11	1.17
ETC	0.61	0.59	1.36	1.41
KNN	0.08	0.09	0.17	0.19
LSTM	121.66	116.91	145.22	148.21
CNN	90.07	123.70	127.02	111.87
CNN-LSTM	102.81	158.38	211.47	215.01

**Table 14:** Comparison results with previous studies.

Reference	Model	Accuracy	Precision	Recall	F1 Score
[17]	RF	0.97	0.97	0.97	0.97
[24]	LSTM	0.997	0.995	0.995	0.995
This Study	ETC	1.00	1.00	1.00	1.00
	ADA	1.00	1.00	1.00	1.00
	KNN	1.00	1.00	1.00	1.00
	RF	1.00	1.00	1.00	1.00

**Table 15:** Statistical analysis using *t*-test.

Case	T-Score	CV	Null Hypothesis
ML using Original Features vs. ML using 60 Features	6.23	6.63 $\times \;10^{-17}$	Reject
ML using 50 Features vs. ML using 60 Features	1.7	6.63 $\times \;10^{-17}$	Reject
ML using 70 Features vs. ML using 60 Features	1.7	6.63 $\times \;10^{-17}$	Reject

## Data Availability

Not applicable.
